# Design and Study of Fractal-Inspired Metamaterials with Equal Density Made from a Strong and Tough Thermoplastic

**DOI:** 10.3390/polym15122650

**Published:** 2023-06-12

**Authors:** Levente Széles, Richárd Horváth, János Péter Rádics

**Affiliations:** 1 Doctoral School on Materials Sciences and Technologies, Óbuda University, H-1034 Budapest, Hungary; szeles.levente@cl.uni-obuda.hu; 2 Bánki Donát Faculty of Mechanical and Safety Engineering, Óbuda University, H-1034 Budapest, Hungary; radics.janos@bgk.uni-obuda.hu

**Keywords:** fractal-inspired geometry, lattice structure, finite element method, additive manufacturing, compressive behavior, energy absorption

## Abstract

In this study, we created metamaterials consisting of square unit cells—inspired by fractal geometry—and described the parametric equation necessary for their creation. The area and thus the volume (density) and mass of these metamaterials are constant regardless of the number of cells. They were created with two layout types; one consists solely of compressed rod elements (ordered layout), and in the other layout, due to a geometrical offset, certain regions are exposed to bending (offset layout). In addition to creating new metamaterial structures, our aim was to study their energy absorption and failure. Finite element analysis was performed on their expected behavior and deformation when subjected to compression. Specimens were printed from polyamide with additive technology in order to compare and validate the results of the FEM simulations with real compression tests. Based on these results, increasing the number of cells results in a more stable behavior and increased load-bearing capacity. Furthermore, by increasing the number of cells from 4 to 36, the energy absorption capability doubles; however, further increase does not significantly change this capability. As for the effect of layout, the offset structures are 27% softer, on average, but exhibit a more stable deformation behavior.

## 1. Introduction

The behavior of metamaterials can be characterized and investigated in terms of several mechanical [1,2,3,4], thermal [5,6], and even vibrational [7,8,9,10,11] parameters. Of course, depending on the structure, especially if (but not exclusively) auxetic materials are the subject of investigation, metamaterials can also be characterized by Poisson’s ratio [1]. If the metamaterial structures are appropriately parameterized or the right structure is designed, it is possible to impart several advantageous properties to the structure simultaneously. 

At the turn of the millennium, Deshpande et al. [12] discussed the topological criteria that dictate whether a structure is dominated by bending or stretching, which affects the deformation behavior of the metamaterial. Chen et al. [13] later observed this phenomenon. They examined three-dimensional lattice structures and found that the resistance to compression of the bending-dominated Kelvin structure is almost half of the value measured in case of the octet or cuboctahedron structure. In general, bending dominant structures can absorb more energy [14]. However, Wagner et al. [15] were able to bridge the gap between bending and stretching-dominant behaviors with a new type of structure that can change its behavioral mechanics upon external (heat) influence. Hierarchical stretch–bend hybrid structures were created in 2013 [16], where the elementary cell had a pyramidal geometry. 

Furthermore, Al Nashar M. and Sutradhar A. [17] created hierarchical lattice structures in order to investigate their superior energy absorption. The authors found that with the introduction of hierarchy, the energy absorption capacity of the structure increased by a factor of 4–5.

Besides hierarchical structures, “compression–torsion” behavior has received considerable attention. In 2019, Zhong et al. [18] investigated a three-dimensional structure consisting of parallel planar planes with simple square cut-outs, in which an inclined rod element connected the corners of the squares to produce torsional behavior under compression. A similar geometric design methodology was used by Zheng et al. [9]—instead of the simpler square cut-outs, the horizontal parallel planes were tetra-chiral-based. They found that increasing the number of elements only increases the TTC (tension–torsion-coupling) effect for a while, which, depending on the structure, can decrease as the number of elements increases. In the above-mentioned two publications, the horizontal planes were coupled at the corner points with one inclined rod element; thus, in the structure created by Wang et al. [19], each corner is connected with two inclined rod elements. Of course, the structure still exhibits the compression–torsion behavior, and the authors designed an even more efficient structure by eliminating the redundant regions.

A structure can be optimized not only for a compressive load in one direction, but also for a two- or three-directional load. Along these ideas, Ren et al. [20] extended the concept of the “buckling-based negative stiffness” lattice from one direction to two or three directions. Conventional and auxetic structures do not need to be considered separately, since with suitable paring, the combination results in a geometry with higher energy absorption [21]. In addition to conventional and auxetic metamaterials, Wu et al. [22] created a new family of structures that respond to both compressive and tensile loads with expansion. 

Three completely new “compression-induced twisting” three-dimensional structures were created by Li et al. [23] inspired by the shear–compression coupling effect. The sides of the novel 3D elementary cells were given by two-dimensional shear–compression coupling effect–based geometries. Besides the conventional structures, auxetic unit cells were also used to create structures with compression–torsion behavior [24].

Mechanical properties can also be improved with simple rod elements, for example stiffening the Tetra-missing rib honeycomb structure. Zhu et al. [2], in their work, not only examined the effect of stiffening but presented a parameter-based analysis. The modified structure resulted in improved mechanical properties. 

Another way to improve the mechanical properties of metamaterials is to change the number of elements. This was investigated by Carneiro et al. [25] for auxetic metamaterials. The most practical way to vary the number of cells to make the results comparable is for each specimen to have the same mass/relative density. Hend et al. [26] performed a comparative study of TPMS (triply periodic minimal surface) lattice structures. They concluded in this comprehensive study of 18 specimens that the resistance of the specimens to compression is most influenced by the structure and the size of the elementary cell. It is also evident from the study that the relative density and the size of the elementary cells have an effect on the mechanical properties of the specimens. Yang et al. [27] investigated these two effects in parallel. Increasing the number of elementary cells (while the mass of the specimen remained the same) increased the compaction resistance and the energy absorbed by the specimens. Increasing the relative density is also advisable, since stress peaks are less likely to occur and are less significant in thinner regions, and the yield force also increases [28]. The above-mentioned results on the number of cells and relative density are also supported in more recent studies [29]. It is also possible to achieve better mechanical properties by varying not only the size but also the aspect ratio of the elementary cells [30]. Cell orientation can also affect mechanical properties, by shifting the deformation to stretching-dominant from bending. [31]

In addition to the traditional geometric and structural changes, there are also different ways to create lattice structures with more favorable properties. Tan et al. [32] created a structure whose energy absorption can be interpreted not only in the elastic but also in the plastic zone, under repeated loading. More favorable properties can be imparted to the metamaterial with the continuously increasing space filling of the individual layers (so-called gradient design), while the element cell geometry remains the same. Zhang et al. [33], for example, observed a significantly better energy absorption capacity in this case. Charles M. et al. [34] also found that in certain instances the specially graded design is more favorable for impact resistance. New lattice structures can also be created by combining two of the most dynamically developing technologies of our time, namely, additive manufacturing technologies with topology optimization [35]. Finally, a given geometry can only exhibit the desired behavior if the constituent material allows it, since with different materials, the behavior of the structures studied differs significantly [36].

Geometry-based modifications are presented above, but one can draw inspiration from other areas of science. Fractals formed the inspiration for our research geometry; fractals, whose history goes back a long time, are infinitely complex patterns that are self-similar [37,38]. One might recognize fractals from the best-known geometries: the Koch snowflake [38], the Sierpinski gasket [39,40], and the Sierpinski sponge [41,42].

Not only are the methods for improving the properties of lattice structures extremely diverse, but also their applications. The outstanding energy absorption capabilities of lattice structures are implemented in blast-resistive structures [43], crash boxes [44,45], passive safety elements such as helmets [46], car parts [47], and even biomedical applications [48]. The tunable and designable properties of lattice structures make them compelling for space applications [49] to achieve parameters that cannot be satisfied by traditional manufacturing methods. 

The contemporary literature presented above contains several methods for stiffening metamaterials and achieving favorable properties, which have proven to be very effective; however, their geometry is very complex. The benefits of newly developed and improved lattice structures are truly demonstrated at the professional, industrial level; however, bringing complex real objects filled with complex structures to life (via additive manufacturing) is quite a challenge. Thus, we came to the conclusion that a thorough study of the simple geometries presented in this paper, made from advanced, tough, and durable thermoplastic on an industrial powder-bed (SLS) printer, could benefit the industry more broadly.

In this paper, the effects of two methods for stiffening metamaterials, thereby achieving better mechanical properties, are analyzed on a simple structure. Fractal-inspired specimens with simple square cut-outs subjected to compression testing were prepared with increasing element number at the same mass, providing an opportunity to evaluate the effect of element number. The other effect under investigation was the effect of geometry layout; each specimen was prepared in two layouts, one ordered and one centrally offset. 

## 2. Materials and Methods

Our aim is to describe a simple geometry of a repeated elementary cell of a fractal-inspired lattice, whose cross-section, and therefore its volume and mass, do not change as the number of elements increases along given enclosing dimensions. 

The limit to increasing the number of elements under macro-conditions is imposed only by the limits of the particular additive manufacturing technology. However, their geometry, like fractals, can be considered infinite patterns.

### 2.1. Mathematical Description of the Geometry of Metamaterials

**Figure 1:**
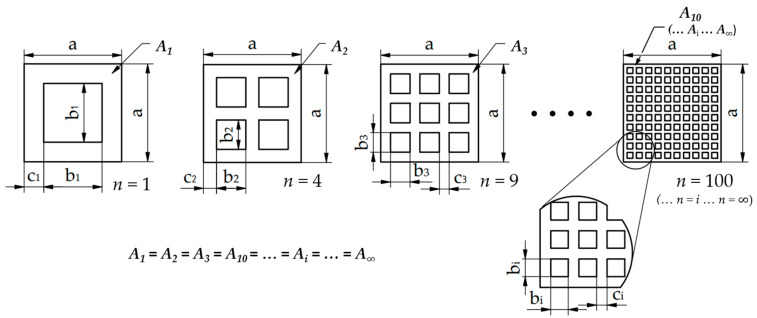
Derivation of a metamaterial with equal cross-sections and equal enclosing dimensions (where: *a*—the side of the enclosing square of the metamaterial; *b*_1_—side of the square piece of material cut out of a specimen consisting of 1 element; *b*_2_—side of the square piece of materials cut out of a specimen consisting of 4 elements; *b*_3_—side of the square piece of materials cut out of a specimen consisting of 9 elements, and *b_i_*—side of the square piece of material cut out of a specimen consisting of *i* cells, (elements along one side of the enclosing square) ($i=\sqrt{n}$); *n*—number of elements; *c_i_*—thickness of the remaining material section for a specimen consisting of *i* cells); *A*_1_, *A*_2_, *A*_3_*… A_i_*—cross-sections of metamaterials; *V*_1_, *V*_2_, *V*_3_… *V_i_*—volumes of metamaterials).

The basic idea for deriving a self-repeating metamaterial with the same cross-section was provided by the Sierpinski sponge; some of its steps are shown in Figure 1.

Below, we describe the derivation of metamaterial cross-sections from each other. As an example, specimens consisting of 1, 4, and 9 elements are presented. Finally, the general form of derivation is given.

(1) $$A_{1}=a^{2}-b_{1}^{2}$$

(2) $$A_{2}=a^{2}-4\cdot b_{2}^{2}$$

(3) $$A_{3}=a^{2}-9\cdot b_{3}^{2}$$

Assuming that *A*_1_ = *A*_2_ = *A*_3_ = *A_i_*, the dimensions of the specimen consisting of 1 cell can be used to determine the dimensions of the material sections cut out for additional metamaterials. For a 4-element cell:

(4) $$A_{1}=A_{2}$$

(5) $$a^{2}-b_{1}^{2}=a^{2}-4\cdot b_{2}^{2}$$

(6) $$b_{2}=\frac{b_{1}}{2}$$

Similarly for a 9-element metamaterial:

(7) $$A_{1}=A_{3}$$

(8) $$a^{2}-b_{1}^{2}=a^{2}-9\cdot b_{3}^{2}$$

(9) $$b_{3}=\frac{b_{1}}{3}$$

Consequently, any *n*-element metamaterial can be described as follows:

(10) $$A_{1}=A_{i}$$

(11) $$a^{2}-b_{1}^{2}=a^{2}-n\cdot b_{i}^{2}$$

(12) $$b_{i}=\frac{b_{1}}{\sqrt{n}}$$

In the light of the foregoing, the spatial extrusion of the constant cross-section metamaterials results in constant volumes; thus, regardless of the element number, it follows that:

*V*_1_ = *V*_2_ = *V*_3_ = … = *V_i_* (13)

In evaluating the results, it is advisable to consider the slenderness of the material sections constituting the specimens. First, the thickness of the constituent material sections must be determined, which can be described with the following equation:

(14) $$c_{i}=\frac{a-(\sqrt{n}\cdot b_{i})}{\sqrt{n}+1}=\frac{a-(\sqrt{n}\cdot \frac{b_{1}}{\sqrt{n}})}{\sqrt{n}+1}=\frac{a-b_{1}}{\sqrt{n}+1}$$

The slenderness ratio for these metamaterials is as follows:

(15) $$\frac{b_{i}}{c_{i}}=\frac{\frac{b_{1}}{\sqrt{n}}}{\frac{a-b_{1}}{\sqrt{n}+1}}=\frac{b_{1}\cdot (\sqrt{n}+1)}{\sqrt{n}\cdot (a-b_{1})}$$

As the number of elements increases, the slenderness ratio will tend to a limit (see Figure 4).

### 2.2. Introduction of the Two Studied Metamaterial Structures

Part of the literature reviewed in the introduction chapter examined methods of stiffening the structure and controlling the deformation behavior of metamaterials. The reviewed literature contained complex structures and modeling techniques, which cannot be applied easily in all cases.

In this paper, two structures (consisting of the same element on the micro level) created based on the principles presented in Section 2.1 are examined with a different number of cells, with an ordered and a centrally offset design at the macro-level. The reason for this is that, because of compressive load, it is expected that in the ordered geometry only compressed bars will be load-bearing (at least at the first phase of the loading). In the offset geometry, however, the horizontal regions are immediately subjected to bending.

**Figure 2:**
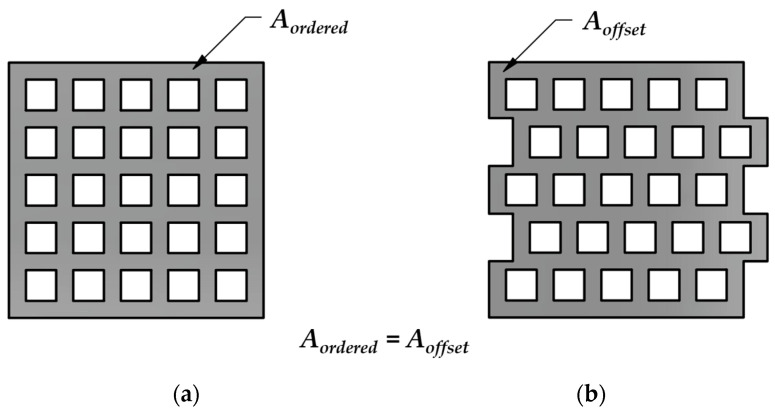
Theoretical presentation of ordered and offset specimens with shifted cells (hereinafter “offset geometry”) (for *n* = 25 cells). (**a**) Ordered; (**b**) offset.

In the case of the ordered structure shown in Figure 2a, it is foreseeable that the deformation response to the compressive load is neither predictable nor controlled. As the load progresses, the deformation in the cut-out regions (regions with no material) result in macro-level buckling, i.e., the specimen “collapses”.

High-performance metamaterials must exhibit controlled and predictable behavior. The fundamental idea behind creating the centrally offset structure was based on this guideline.

Owing to the novel structure, the path of the force is broken, and the entire structure can deform along the mutual deformation of each cell at the macro-level without significant lateral deflection. The proposed offset arrangement provides better behavior and properties at the same weight without complex geometrical modifications.

The modified—offset—structure is expected to present more favorable results in terms of the force required for compression and the energy absorbed. The emergence of bending stress in the horizontal material sections is anticipated to result in a more uniform stress distribution in the geometry.

Based on the equations presented earlier we created the metamaterials in CAD with the following numbers of element: *n* = 1, 4, 9, 16, 25, 36, 49, 64, 81, and 100.

**Figure 3:**
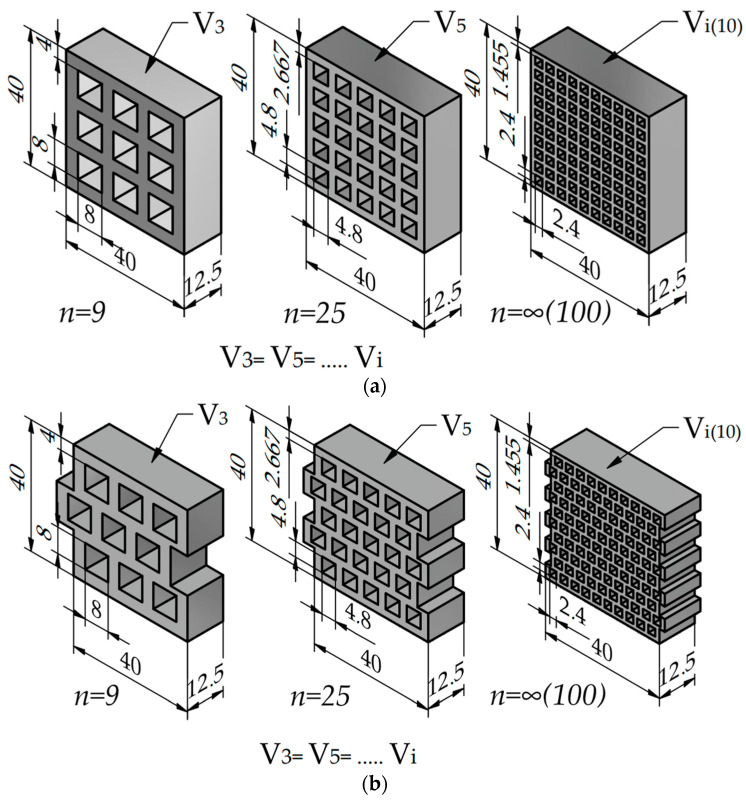
Photo-rendered images of modeled metamaterials (*n* = 9, 25, 100). (**a**) Ordered geometries; (**b**) offset geometries.

The side of the enclosing square is *a* = 40 mm for all of the specimens and *b*_1_ = 24 mm. (the theoretical weight is 12.2 g in all cases). Figure 3 shows the CAD rendering of some of the specimens.

Based on the dimensions of the models used in this study, a limit value calculation was performed by substituting the constants “*a*” and “*b*_1_” into Equation (15). The slenderness ratio tends to the limit of 1.5.

(16) $$\underset{n\to \infty }{\lim}\left(a=40;b_{1}=24\right)=\frac{b_{1}\cdot (\sqrt{n}+1)}{\sqrt{n}\cdot (a-b_{1})}=1.5$$

**Figure 4:**
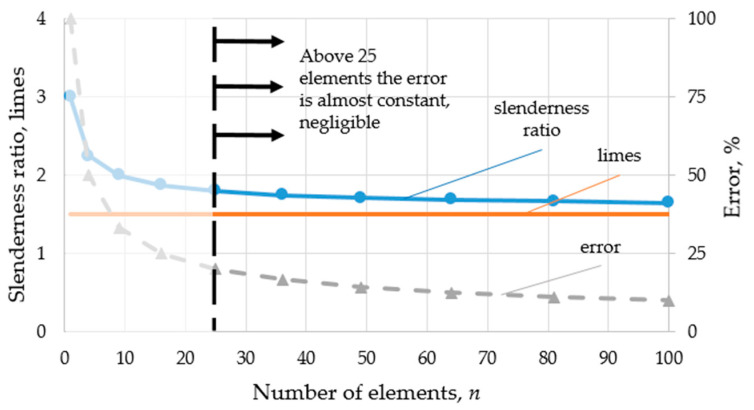
Slenderness ratio as a function of element number.

The function is plotted in Figure 4. It can be concluded that in this case, the deviation from the limit above 25 elements is small and decreases moderately as the number of elements increases. Thus, above a certain number of elements (25 in our case), the slenderness ratio can be considered almost constant, which is expected to be reflected in the compression tests.

### 2.3. Estimation of Macrostructural Behavior Based on Microstructural Constituents

**Figure 5:**
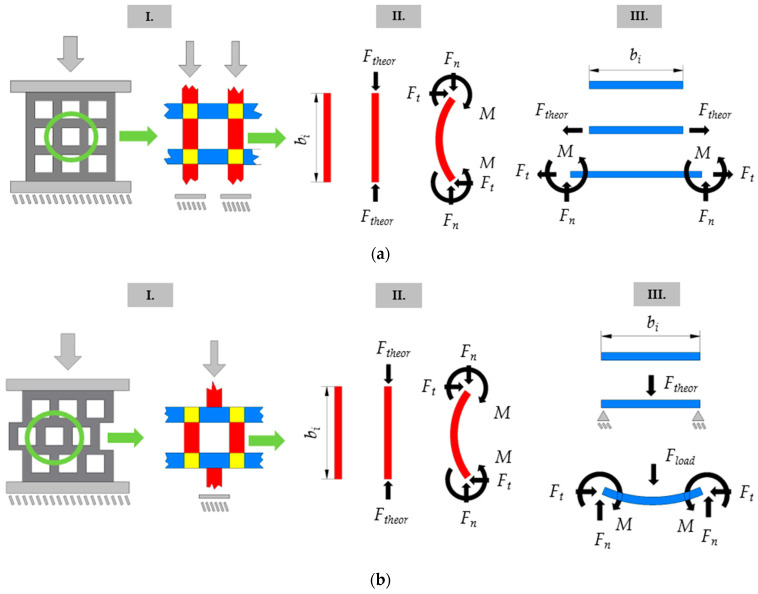
Component breakdown; the expected behavior of components. (**a**) Ordered geometry; (**b**) offset geometry (where: I. Pre-load condition; II. Theoretical loads—at the exact moment of loading; III. Loads on the expected deformed shape; *b_i_*—dimension of the material section cut out for the specimen consisting of *i* number of cells; *F_theor_*—theorical force at the moment of loading; *F_load_*—transfer load from metamaterial segments; *F_t_*—tangential reaction force; *F_n_*—normal reaction force; *M*—load moment).

The behavior at the macrostructural level of the different structures (ordered and offset) can be explained by breaking down the specimens into components as detailed below and shown in Figure 5. In all cases, the boundary of the component breakdown is formed by regions with different elementary loads. The breakdown for the studied structures results in horizontal (blue), vertical (red), and nodal (yellow) elements. The expected theoretical deformation behaviors are shown in a simplified manner in Figure 5. 

In the ordered structure (Figure 5a), the vertical components are subjected to pure compression, while the horizontal regions are subjected to pure tension. The compressive regions are more significantly loaded than the tensioned regions, especially in the initial stage of deformation; thus, the geometry is not loaded/utilized evenly. As the deformation progresses, buckling of the compressed regions is expected, resulting in tensile and torsional loads in the horizontal (blue) components through the nodes. 

In the offset structure, however, we cannot speak of purely tensioned components, since bending is already present at the initial stage of the deformation load (Figure 5b). The vertical (red) components transfer the compressive load directly to the center of the horizontal components. The result is a simply supported beam with a central compressive (bending) load. Naturally, in the case of the offset structure, the buckling of the vertical (compressed) regions will occur as the load progresses. Figure 5 also shows the horizontal and vertical components forming the specimens in three cases. In the figures, the equilibrium condition at rest—before loading—is followed by a theoretical representation of the loads at the exact moment of loading. Finally, an expected deformation behavior is shown, alongside the acting forces and moments.

### 2.4. Manufacturing Metamaterials with Additive Technologies

The specimens were made from polymer with powder-bed SLS technology. The specimens were prepared on a 3D Systems ProX 6100 machine (3D Systems Corporation, Rock Hill, CA, USA) horizontally oriented in the build volume, using DuraForm ProX Pa material. The polymer powder was removed from the internal cavities and the surfaces of the specimens once printing was completed.

### 2.5. FEM Test Environment

In addition to real-life compression tests, the additively manufactured specimens were also subjected to finite element analysis. Our aim was to determine the usability and accuracy of the finite element method for the developed metamaterials. For the comparison of the real-life compression tests and the finite element method, we first had to create a finite element environment that describes the real mechanical conditions. ANSYS Workbench 2022 R1 software was used for the FEM simulations. Considering the slow speed of compression, the Static Structural module of ANSYS Workbench was selected to perform the tests. 

**Figure 6:**
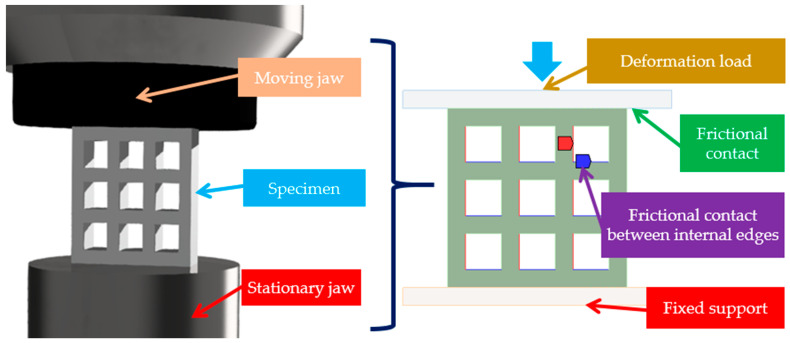
The real test environment and its FEM representation.

The first step in finite analysis is to pre-process the geometry. Considering the structure of the specimens, two-dimensional (plane stress) simulations are sufficient, which reduces calculation time. During compression testing, the specimens are placed in between the stationary and moving jaws of the compression machine. For a sufficiently accurate representation of the expected behavior, both the moving and the stationary jaws of the compression machine form part of the two-dimensional FEM model (Figure 6). 

### 2.6. Boundary Conditions and Loads in the Finite Element Environment

In accordance with the real compression conditions, the lower edge of the stationary jaw is rigidly fixed, and translation and rotation are not allowed. The load is deformation-based, and the simulation is considered up to the moment of compaction (unlike real compression tests) due to the characterization of the metamaterial (as opposed to the characterization of a solid material). The load exerts its effect on the upper edge of the moving jaw. Due to the relatively high deformation load, a non-linear analysis approach is required. For a clearer evaluation of the results and a more certain convergence with respect to non-linearity, the deformation load is applied in steps (20 steps—incremental load).

In addition to geometrical non-linearity (essentially parallel to it), contact non-linearity also occurs. As a result of the relatively large deformation (almost half the height of the specimen), the edges of the metamaterial come into contact with each other, rest on each other, and then frictionally slide on each other as deformation progresses. In accordance with the above, it is necessary to model the interaction of edge pairs that can potentially come into contact with each other. The behavior can be approximated in a finite element environment by specifying frictional contact pairs and setting the friction coefficient to 0.35 between the potentially contacting edges or edge segments. The frictional contact regions, edge pairs, or edge segment pairs must always be specified individually based on the specific behavior of the specimen. There is also a frictional contact between the jaws of the compression machine and the specimen. The coefficient of friction here is 0.2 for steel–polyamide contact [50] (see Figure 6).

It was important that at least 3 elements were placed along each cross-sectional thickness in the finite element mesh. Quadrilateral dominant meshing method was implemented with linear element order. As deformation progresses, the studied specimen and the mesh are significantly distorted, which is why we used the adaptive remeshing module for the specimens. Due to geometrical non-linearity, large deformation was allowed for the solver. 

### 2.7. Material Model

The non-linear nature of the problem was mentioned in the previous sections, which is also reflected in the material model. With the data provided by the manufacturer, the non-linear behavior of the material can be established. Therefore, material behavior is best approximated by a multilinear isotropic hardening model. The density required to set up the material model can be obtained from the manufacturer’s data sheet, while the necessary stress–strain values can be obtained from previous research results [51].

## 3. Results 

### 3.1. Compression Test Results

**Figure 7:**
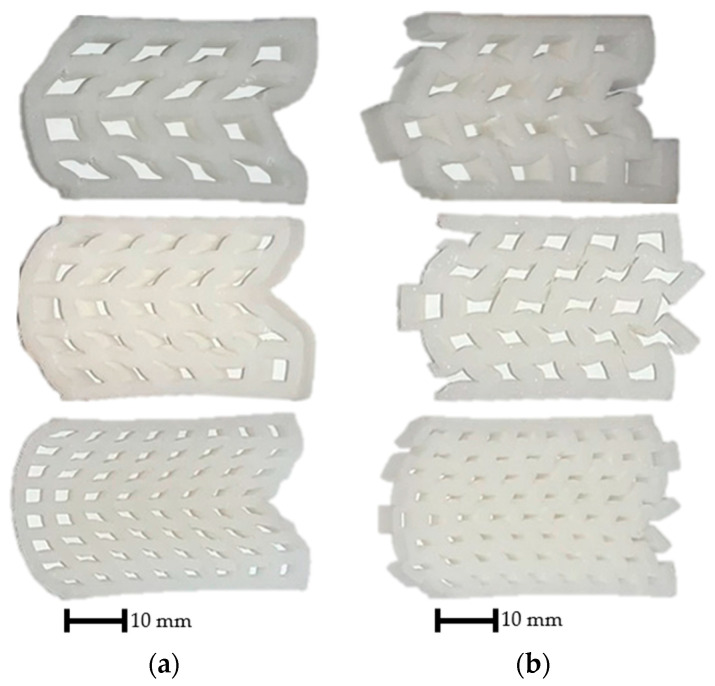
Specimens after the compression tests (*n* = 16, 25, 64). (**a**) Ordered geometries; (**b**) offset geometries.

The specimens were compressed on a Hegewald & Peschke 40-ton machine. The compression speed was 5 mm/min. The test lasted until a deformation of 20 mm was reached (50% deformation) or until complete failure. Figure 7 shows some typical deformations. Cracks and fractures appear on the specimens as a result of compression. The more elements the geometry consists of, the smaller the extent and the significance of the cracks. The effect of geometrical offset can clearly be observed in the deformed shapes; the ordered geometries always tilt sideways (Figure 7a), while the offset geometries deform in a much more ordered manner, i.e., they collapse in on themselves (Figure 7b). 

**Figure 8:**
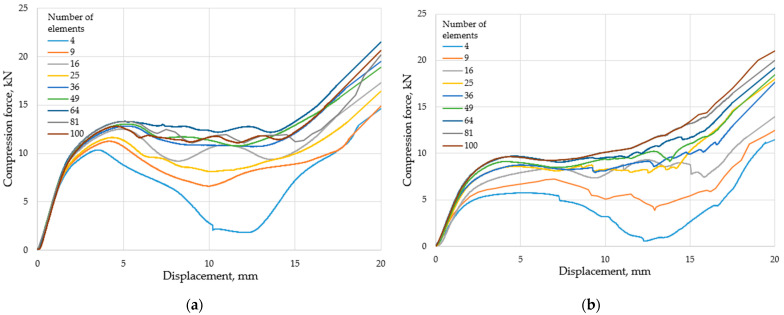
The result of the compression tests. (**a**) Ordered specimens; (**b**) offset specimens.

The compression tests were therefore designed to show the force–displacement behavior of each specimen. The force–displacement curves of Figure 8 clearly show the non-linear behavior and the failure of specimens. The force peaks appearing on the curves followed by a significant decrease clearly show the failure of certain regions, i.e., rows. 

In the case of the examined polymer specimens, regardless of the structure, a significant increase in the absorbed force can be observed towards the end of the deformation load. This increase is the result of more and more regions and edges resting on each other, in other words, the increase is the result of the increasing compaction.

Stresses in the ordered specimens (Figure 7a) cause these specimens to crack at the corners, although the effect, size, and number of these cracks decreases as the number of elements increases. The phenomenon is perfectly illustrated in the case of the four-element specimen; following the initial force peak, there is a significant and sudden drop in the compression test results (Figure 8a). As the specimens crack, their resistance to load also decreases. This phenomenon is less relevant with specimens consisting of more elements, and for the specimens with 100 elements there is almost no such thing as a drop in the force values as the deformation progresses. 

As a result of compression, cracks also appear in the offset specimens (Figure 7b), which is also reflected in a drop in the force–displacement curve (Figure 8b). The drop is only present for the 4-element and 9-element specimens (and to a small degree, the 16-element specimen). Owing to the bending and compressive load, the offset structure is more stable than the ordered structure. The load resistance of the regions subjected to bending is lower than that of the compacted regions, so the initial peak force value of the curves is also smaller. 

Not only do the cracks truly explain the drops in the curves, but also the combined effect of the geometrical parameters and the forming cracks. Specimens with a higher slenderness ratio are more prone to buckling, resulting in lower resistance against the load. Figure 8 shows that specimens consisting of 25 or less elements exhibit the drop in the force–displacement curves. Due to buckling, the local deformation and stress at the sharp corners ultimately result in material failure and crack forming. Thus, the effect of the slenderness ratio (Equation (15)) derived in Section 2.1 is unambiguous, since the function has a limit value, which is already sufficiently approximated by the specimen of 25 elements. Hence, for specimens of 36 or more elements, the decrease in force in the force–displacement curve is hardly noticeable.

Due to the fractures and cracks in the specimens, finite element simulations only give meaningful results in cases with 36 or more elements. Figure 8 shows that the compression path region purely characteristic of the metamaterial lasts up to 15 mm, after which compaction can be observed in the upward phase. Therefore, the characteristic behavior of the metamaterials presented in this study is in the 0–15 mm compression range; thus, the finite element tests were also carried out for this region.

The force–displacement values for the FEM results were obtained by applying a probe to the lower edge of the mowing jaw (see Figure 6), following the nature of the real compression tests. 

**Figure 9:**
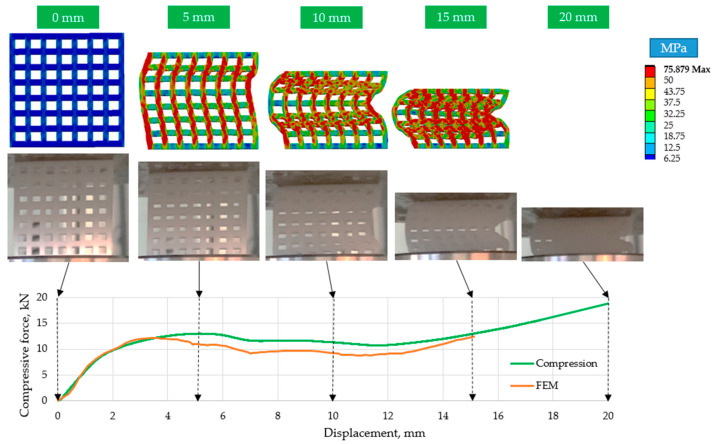
Comparison of the finite element simulation results with the real compression tests for *n* = 49 elements.

**Figure 10:**
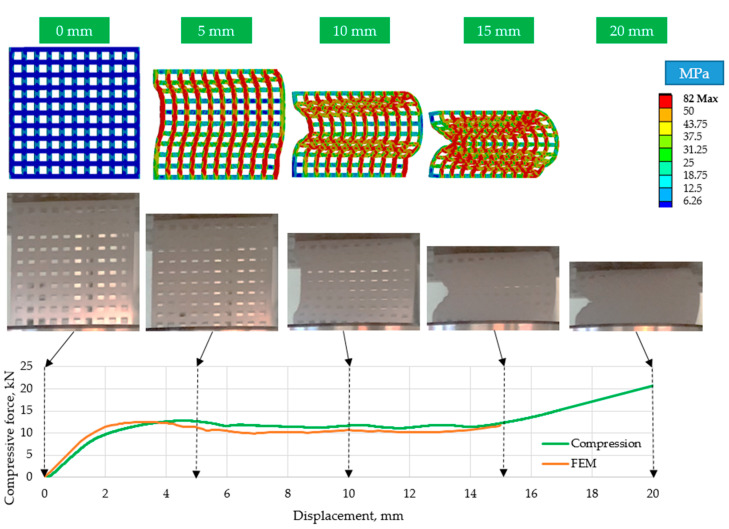
Comparison of the finite element simulation results with the real compression tests for *n* = 100 elements.

Figure 8 shows that 15 mm (37.5%) compression results in an almost completely compacted (solid) specimen. The ordered structures were unstable; they buckled laterally (Figure 9 and Figure 10). As can be seen for two typical cell numbers (*n* = 49 and *n* = 100), the specimens deformed in different directions, proving the unplanned behavior of the structure (the results were similar for all the ordered specimens). 

**Figure 11:**
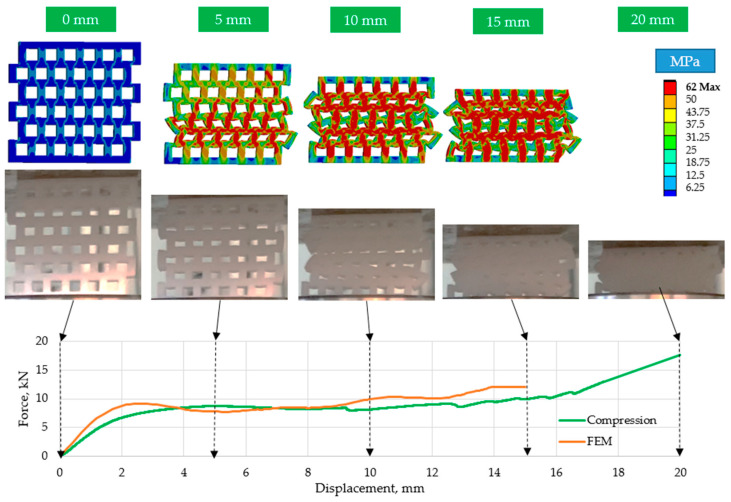
Comparison of the finite element simulation results with the compression tests for *n* = 36 offset elements.

Figure 9, Figure 10 and Figure 11 show that the curves of the finite element simulation and the real compression tests do not differ significantly. The area under the curves, which characterizes the energy absorbed through compaction, only differs slightly, indicating the applicability of the finite element method for relatively large deformation behavior with high accuracy.

During the compression, the offset specimen’s more structured self-collapsing deformation behavior can be inspected in Figure 11. The deformation behavior and the force–displacement curve are also perfectly shown by the finite element method for these structures. 

### 3.2. Assessment of the Absorbed Energy and Comparative Analysis of Offset and Ordered Structures

In addition to the deformation behavior, the specimens studied can be characterized by the energy absorbed through compression. The energy absorbed (in the 50%, or 20-mm, deformation range) is defined by the area under the curves in Figure 8.

(17) $$E_{absorbed}=\int_{0}^{20}f\left(x\right)dx$$

In Equation (17), *f*(*x*) refers to the force–displacement curves, where the integral was considered for the 0–20 mm compression range. In our study, the absorbed energy was determined numerically based on the principal of Equation (17). Table 1 shows the absorbed energies.

Table 1 shows that specimens/metamaterials with an offset geometry can absorb less energy in all cases. 

Table 1 also shows that the absorbed energy does not correlate with the number of elements for the ordered specimens, due to the unstable buckling behavior, which is a result of the vertical segments subjected solely to compression. On the other hand, for the offset geometry, the absorbed energy increases monotonically, owing to the predictable (non-buckling) deformation behavior. This is presumably related to the slenderness ratio. Following the presented test and evaluation methods, the behavior of more complex fractal geometries can be studied; one can even consider our work as a simplified model for fractal materials.

## 4. Conclusions

In this study, a family of easily applicable metamaterials with a simple fractal-inspired structure is presented along with the fundamental tests and studies, which help to understand their behavior. In contrast to more complex metamaterials, the presented structures are easier to integrate into an actual design, thus further improving the benefits of industrial 3D printers, by using less material and providing designable characteristics. The derived mathematical principles allow the reader to construct the structure with any number of elements (maintaining the same surface, same volume, and same mass, regardless of element number). In this study, the effect of geometrical offset compared to the ordered structure was investigated. The specimens were made from the special DuraForm ProX PA material, a durable, long-lasting, high-performance material. Based on our investigation, the following conclusions can be drawn:

- Increasing the element number results in a more stable behavior, i.e., the load-bearing capacity increases throughout the entire deformation load. The behavior of the specimens can be considered stable for 36 or more elements.
- As the number of elements increases, the absorbed energy also increases for both structures studied; increasing the element number from 4 to 36 almost doubles the amount of energy absorbed. Increasing the element number above the stable threshold (36) does not significantly increase the energy absorption capability.
- Since the ordered structure is composed purely of compressed components, its load-bearing capacity and absorbed energy rate are also higher. At the same time, the results of the tests show that the deformation behavior of the offset structure is predictable and designable in comparison with the ordered structure.
- Offset structures are softer; on average, 27% less force is required to compress them.
- The finite element simulations also suggest that the stress distribution of the offset specimens is more uniform.
- The finite element method showed accurate and reliable results above 36 elements and can therefore be applied under the presented conditions.

## Figures and Tables

**Table 1:** Energy absorbed by the tested metamaterial geometries.

Number of Elements, *n*	Absorbed Energy (Ordered Geometry), J	Absorbed Energy (Offset Geometry), J
4	131.5	81.6
9	169.7	119.0
16	206.9	155.5
25	189.1	174.1
36	226.4	172.2
49	230.5	188.2
64	250.0	201.3
81	236.0	209.8
100	234.1	215.0

## Data Availability

The data presented in this study are available on request from the corresponding author.
